# LONG COVID: IT’S A BIG DEAL

**DOI:** 10.1093/geroni/igad104.2053

**Published:** 2023-12-21

**Authors:** Laura Mehegan, Rohini Khillan, Erwin Tan, Olivia Dean, Teresa Keenan, Lona Choi-Allum, Jim Palmieri

**Affiliations:** AARP, Washington, District of Columbia, United States; AARP, Washington, District of Columbia, United States; AARP, Washington, District of Columbia, United States; AARP, Oakland, California, United States; AARP, Washington, District of Columbia, United States; AARP, Washington, District of Columbia, United States; AARP, Washington, District of Columbia, United States

## Abstract

AARP fielded two nationally representative surveys of adults aged 50 and older to explore their experiences with COVID and long COVID (lingering symptoms for ≥ 4-weeks). The October 2022 survey (N=1,795) was a re-fielding of the May 2022 survey (N=1,018). Survey results showed that 40% of participants were infected with COVID at least once and, among those, 27% experienced long COVID. Among those who experienced long COVID, 33% said it impacted their job. To explore long COVID in greater depth and to understand the financial, health, and social impacts, seven virtual focus groups were held with either long COVID patients (N=27) or partners of long COVID patients (N=20). Focus group participants reported extensive suffering due to debilitating symptoms, most notably brain fog, severe fatigue, depression, and anxiety. Partners reported caregiving duties that impacted their jobs and patients often altered their work schedule or quit their jobs completely. Social isolation was reported in all focus groups. Most participants reported adapting to the loss of income and increased medical expenses; however, the extent of financial consequences is yet to be observed. When queried on the support they needed, most patients and caregivers said they needed more reliable information, and they needed their healthcare providers to share their medical information. It was difficult for patients to understand their symptoms or to get a diagnosis or symptom relief. This research suggests the need to develop and disseminate consistent information on long COVID symptoms and remedies, and to encourage information sharing within the healthcare community.

